# Combined Focused Electron Beam-Induced Deposition and Etching for the Patterning of Dense Lines without Interconnecting Material

**DOI:** 10.3390/mi12010008

**Published:** 2020-12-24

**Authors:** Sangeetha Hari, P. H. F. Trompenaars, J. J. L. Mulders, Pieter Kruit, C. W. Hagen

**Affiliations:** 1 Department of Imaging Physics, Faculty of Applied Sciences, Delft University of Technology, Lorentzweg 1, 2628 CJ Delft, The Netherlands; hari@delmic.com (S.H.); p.kruit@tudelft.nl (P.K.); 2 Thermo Fisher Scientific, Achtseweg Noord 5, 5651 GG Eindhoven, The Netherlands; piet.trompenaars@thermofisher.com (P.H.F.T.); j.mulders@chello.nl (J.J.L.M.)

**Keywords:** lithography, focused electron beam-induced deposition, focused electron beam-induced etching, interconnects, scanning electron microscopy, nanopatterning

## Abstract

High resolution dense lines patterned by focused electron beam-induced deposition (FEBID) have been demonstrated to be promising for lithography. One of the challenges is the presence of interconnecting material, which is often carbonaceous, between the lines as a result of the Gaussian line profile. We demonstrate the use of focused electron beam-induced etching (FEBIE) as a scanning electron microscope (SEM)-based direct-write technique for the removal of this interconnecting material, which can be implemented without removing the sample from the SEM for post processing. Secondary electron (SE) imaging has been used to monitor the FEBIE process, and atomic force microscopy (AFM) measurements confirm the fabrication of well separated FEBID lines. We further demonstrate the application of this technique for removing interconnecting material in high resolution dense lines using backscattered electron (BSE) imaging to monitor the process.

## 1. Introduction

**Figure 1:**
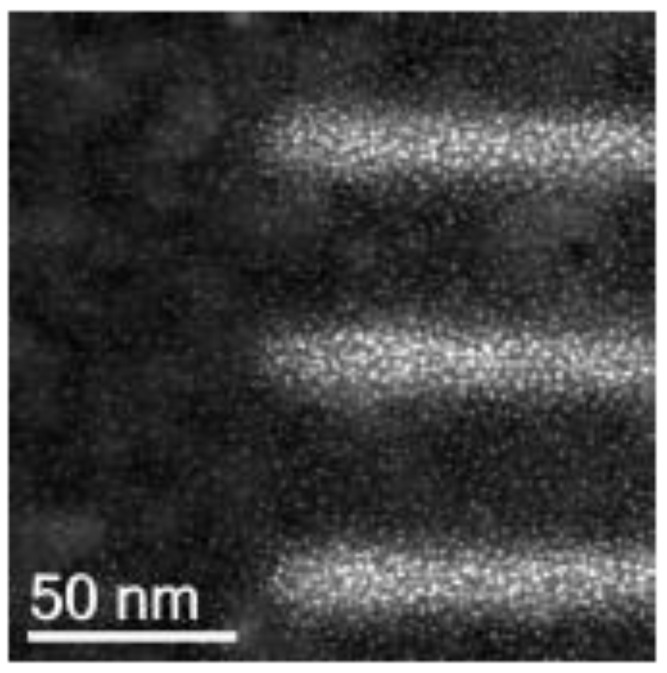
High angle annular dark field (HAADF) image, taken at 80 keV, of dense platinum focused electron beam-induced deposition (FEBID) lines deposited on a 20 nm silicon nitride membrane coated with 20 nm of sputtered silicon and imaged in the transmission electron microscope (TEM). The statistics of the deposition become more evident from the grains visible all the way to the line edges and beyond, giving rise to diffuse deposit in between the lines (private communication M. Scotuzzi and D. Ovchinnikov).

Focused electron beam-induced deposition (FEBID) is a high resolution direct-write nanopatterning technique that can be implemented in a scanning electron microscope (SEM) by scanning the beam in the presence of adsorbed precursor molecules on the substrate. While a myriad of structures such as lines, dots and 3D shapes have been patterned by FEBID, the patterning of sub-20 nm dense lines by FEBID for lithography was only demonstrated recently [1]. As lines are patterned with increasingly lower spacing, they begin to broaden, leading to the question of how well separated they are. This work addresses the issue of interconnecting material in dense FEBID lines. As the scattering of the high energy electron beam in the substrate during FEBID results in the generation of backscattered electron (BSE)’s and SE2′s (secondary electrons generated by BSE’s) over a radius of several hundreds of nanometres, all of which have some probability of dissociating the adsorbed precursor molecules, a thin film of (often) carbonaceous material is deposited over this range. So it is to be expected that in the patterning of dense FEBID lines, this would contribute to the deposition of material in between the patterned areas. As the thickness of this film falls off very gradually, it adds to the challenge of the characterisation of FEBID line profiles, and any measure of line width must somehow take this interconnecting material into account. FEBID patterns have also found several applications in electrical measurements, especially since the development of techniques to improve their conductivity [2,3,4,5,6,7]. There remains, however, the problem of crosstalk due to the halo deposited alongside. This has been demonstrated by Gopal et al. [8] who patterned Pt/C FEBID lines on a Si/SiO_2_ substrate using a range of primary beam energies. They measured a leakage current between them that increased exponentially with increasing beam energy and decreased with line spacing. This observation of the connecting material being conducting is an important result, based on which patterning at lower beam energies appears attractive. However, this is only a solution for lines separated by several microns. As the semiconductor industry approaches the single nanometre regime, the material deposited in between the lines becomes increasingly significant. The transmission electron microscopy (TEM) image of Pt/C FEBID lines deposited on a thin membrane (shown in Figure 1) clearly reveals the presence of interconnecting granular deposits between lines spaced approximately 50 nm apart. Such deposits are a problem not only for electrical measurements but also for shape control.

**Figure 2:**
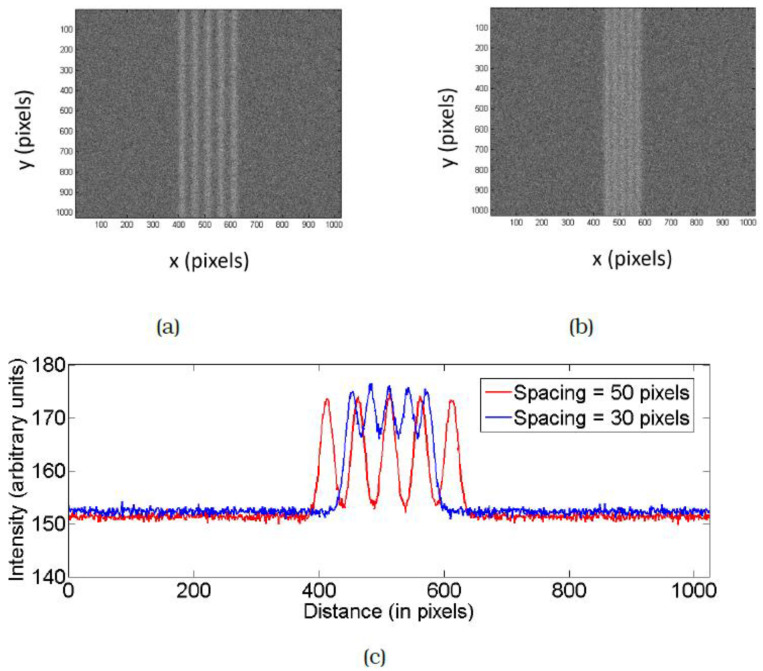
Simulated secondary electron (SE) images of dense FEBID lines with Gaussian profiles. The centre to centre spacing between the lines is (**a**) 50 pixels and (**b**) 30 pixels. (**c**) Comparison of the integrated intensity profile of the two images, clearly demonstrating the increase in amount of material deposited in between the lines as the spacing decreases.

Here we explain how this comes about as a result of FEBID: Figure 2a,b show simulated SE images of two sets of dense FEBID lines at a centre to centre separation of 50 pixels and 30 pixels. The lines were generated as described in [9]. A random signal was generated to have a user-defined power spectral density (PSD). A double Gaussian function was generated with arbitrary parameters and was stretched to match the extent of this signal. This was performed for the left and right edges in each scan line. Poisson noise was added, resulting in the simulated FEBID lines shown in Figure 2. As a result of the linear addition of the Gaussian profiles at the specified separation, the material deposited in between the lines increases with decrease in spacing as is clearly visible from the integrated intensity plot of the two images shown in Figure 2c. This is therefore an unavoidable consequence of patterning lines that have Gaussian profiles. At the values of line separation of interest to us, the cause of this interconnecting deposit (here onwards referred to as interconnects) is expected to be primarily SE1′s (secondary electrons directly generated by the primary electrons), in contrast to the mechanism suggested in Gopal et al. This is because their spatial density is much more than that of BSE’s or SE2′s. SE2 refers to secondary electrons generated not directly by the primary electron beam, but by backscattered electrons. Proximity effects could add to the deposition, causing a line to grow due to electron beam exposure of nearby pixels, resulting in a different integrated profile than the ones shown in Figure 2c. Nevertheless, interconnects could result in a conduction path. One solution would be to pattern lines with vertical sidewalls and no interconnecting tails. However, to do this using FEBID would require complex patterning strategies, not desirable for commercial lithography. Moreover, it is yet to be proven experimentally to be possible at all. Instead, we demonstrate an effective method to remove this connecting material using a one-step technique that can be implemented in the SEM post-FEBID. This has been implemented at high resolution with minimum damage to the line itself. 

The removal of interconnecting material in dense FEBID lines essentially requires a technique that is high resolution, can be implemented inside the SEM, and involves the minimum number of additional steps so that all the advantages of focused electron beam-induced processing are retained. Focused electron beam-induced etching (FEBIE), reported for the first time in 1979 [10] is a chemically selective nanofabrication technique that is complementary to FEBID in that it is top down, with a significant advantage over ion milling due to the absence of sputtering. Adsorbed precursor molecules are dissociated by the electron beam, leading to the formation of reactive fragments which in turn react with the substrate to locally volatilize it. Considering FEBIE in light of the requirements mentioned above, the high resolution of the technique has been demonstrated by [11,12,13]. While the inherent high resolution of the process is apparent as a result of being electron beam-induced, it is to be noted that FEBIE is in some ways more complex than FEBID as a result of surface site activation and secondary reactions involving etch by-products which could govern the process and limit the resolution [14,15,16,17]. Gas-assisted etching has been demonstrated inside a high vacuum SEM with precursor injection through a nozzle [18], as well as in the environmental scanning electron microscope (ESEM) [19,20] where the SEM chamber is flooded with the precursor gas up to pressures of 1–2 mbar while maintaining differential vacuum. While the difference in gas flux in the two cases would alter the surface chemistry somewhat, the processes governing the etching remain the same. Further, as FEBIE involves electron beam exposure in the presence of a suitable precursor, just like FEBID, it involves no post processing, adding only one step to the process. This motivates the choice of FEBIE to effect cleaning in dense FEBID lines, where cleaning refers to the removal of carbon deposit around and in between FEBID structures coming about as a result of the patterning itself.

## 2. Materials and Methods

A large number of FEBID precursors contain carbon, which then often remains in the deposit. A few popular precursors for the deposition of metals are MeCpPtMe_3_, Co_2_CO_8_ and W(CO)_6_, all of which result in deposits containing metal nanocrystallites in a carbonaceous matrix [4,21,22]. The development of a technique that enables the removal of carbonaceous interconnects would be widely applicable and relevant in FEBID, as it would prevent the formation of conducting paths between lines. Based on this, carbon was selected as the material to be deposited. Two commonly used precursors for carbon deposition are naphthalene and phenanthrene, composed of benzene rings. Both precursors have been labelled as carcinogenic and so a better alternative was sought. R.P.G. Siebers and J.J.L. Mulders (unpublished) studied several linear chain alkanes as potential carbon precursors as they also contain only carbon and hydrogen, with the advantage that they are not hazardous to health. Alkanes having 8 to 14 carbon atoms: octane, nonane, decane, undecane, dodecane, tridecane and tetradecane were investigated as they had favourable values of melting point, boiling point and vapour pressure. Larger alkanes were deemed unsuitable as a result of being waxy and therefore very sticky, leading to contamination of the chamber. The lower linear chain alkanes (e.g., pentane, hexane) have a very low sticking coefficient and therefore result in a very low deposition yield. Systematic measurements of deposition yield were made with these alkanes at 5 keV and GIS temperature of 28 °C, based on which dodecane (C_12_H_26_), a liquid at room temperature and not being too contaminating, was selected as the precursor for carbon deposition by FEBID. For the etching of carbon, H_2_O was used. Crystals of MgSO_4_ · 7H_2_O, a hygroscopic material, were loaded into a crucible mounted on a standard gas injection system (GIS). The absorbed water molecules were let in through the nozzle at room temperature. Both FEBID and FEBIE were carried out inside a high vacuum SEM. A Thermo Fisher Helios 650 Dual Beam system fitted with two separate GIS’s for precursor delivery was used. The base pressure in the experiments was in the range of 2 × 10^−6^ mbar to 4 × 10^−6^ mbar. To work in clean conditions, all carbon depositions were performed in succession, after which the chamber was allowed to pump down for at least two hours, and overnight when possible. The etching experiments were then performed in succession.

For lithography, the substrate of interest is silicon. However, the presence of the native SiO_2_ layer presents an issue as it is known to be decomposed under electron beam exposure [23]. This could lead to effects that might interfere with the interpretation of results in this work as we aim to remove deposited carbon alone and not affect the substrate in any way. To prevent this, silicon substrates with a native oxide layer were coated with 20 nm of gold-palladium with a 5 nm titanium adhesion layer. This has the additional advantage of ensuring a strong SE contrast with the carbon deposit due to its significantly higher SE yield.

The patterns were imaged in a Thermo Fisher Helios 650 Dual Beam system fitted with an in-column through-lens SE detector (TLD) and two BSE detectors located higher up in the column (ICD and MD). A standalone Bruker fast scan dimension atomic force microscope (AFM) was used to characterise the height of the patterns before and after etching. Specific locations on the sample were located with the help of pre-fabricated markers [24].

## 3. Results

In comparison to FEBID, FEBIE is complicated by the fact that it is in fact a two precursor system, with hydrocarbons being present in the chamber in addition to the water that has been let in. The partial pressure of these is of course lower than that of water, but the FEBID cross section resulting in the deposition of contamination is higher. So a careful choice of parameters is needed to ensure that etching dominates over the competing FEBID. It seems advantageous to use a high current, increasing the number of SE’s generated. This would increase the number of etching events, while not necessarily increasing the deposition because of the low partial pressure of hydrocarbons. In other words, FEBID of contamination would become precursor limited at a lower current than the etching of carbon, shifting the balance in favour of the latter if the current is increased further. In the interest of extending the results to high resolution experiments, a beam energy of 20 keV was selected as a small spot size can be achieved. 

### Removal of Interconnects

Dense lines of carbon were patterned by FEBID at 20 keV, 3.2 nA, dwell time = 1 s, patterning pitch = 1 nm, number of passes = 1000. Two recipes are described to remove interconnects by FEBIE.

Recipe 1: Large area FEBIE

Cleaning was carried out by exposing a large area containing test patterns made up of dense FEBID lines to FEBIE at 20 keV, 3.2 nA and 10 s dwell time. The dose was determined by observing the SE signal from the etched region and the exposure was stopped when the region in between the lines appeared uniformly bright. The dose needed was found to be in the range from 5 to 300 C/cm^2^.

**Figure 3:**
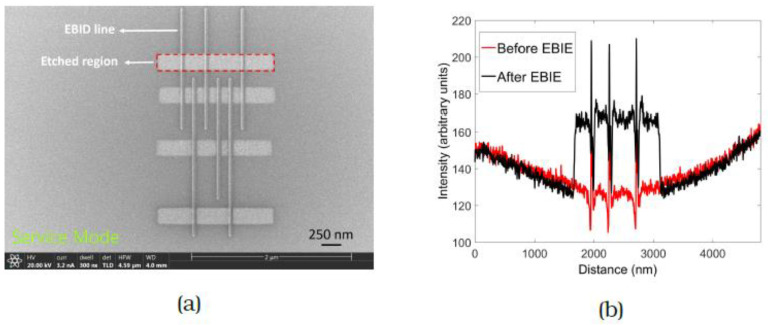
(**a**) SE image of array of carbon FEBID lines after large area cleaning. The red dashed rectangle indicates a typical etched region. Four regions containing lines of spacing 150 nm, 300 nm and 450 nm were etched for a time of 7 s each. (**b**) Integrated intensity plot of topmost region before focused electron beam-induced etching (FEBIE) (red) and after FEBIE (black). The increase in SE signal observed in between the lines demonstrates the removal of carbon and the extent of this region corresponds with the exposed area.

**Figure 4:**
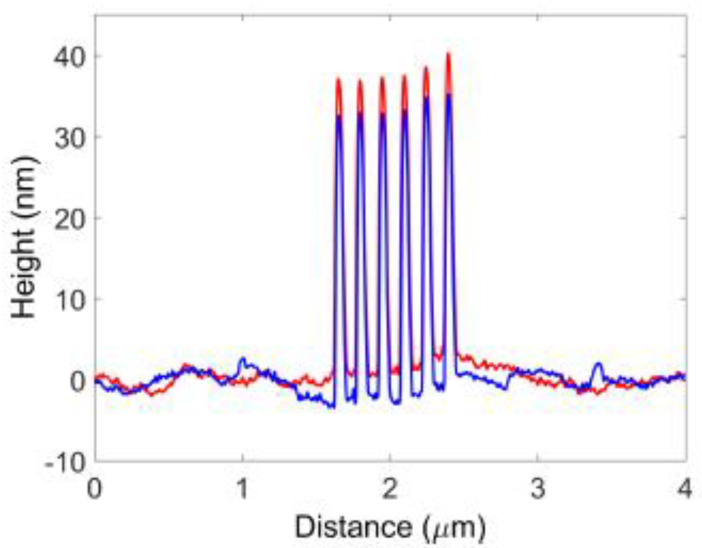
Integrated atomic force microscopy (AFM) height profile taken across the lines shown in Figure 3, which on comparison (Red: as deposited, Blue: after FEBIE) confirm the presence of well separated lines with little or no interconnecting material.

A pattern comprising three arrays of lines spaced 150 nm, 300 nm and 450 nm apart is shown in Figure 3a. These line spacings were chosen to also allow AFM measurements. The red dashed rectangle in Figure 3a indicates a typical etched region containing the FEBID lines. The patterning parameters were 20 keV, 3.2 nA and 10 µs dwell time, with a dose of 18 C/cm^2^. The dose was determined by observing the SE signal from the etched region and the exposure was stopped when the region in between the lines appeared uniformly bright. Etching was performed in four different regions of the FEBID pattern (seen as bright regions in (a)) in serial mode from bottom to top, with one area being cleaned completely before moving on to the next. The removal of interconnecting material is evident from the brightening and the integrated SE intensity plot in Figure 3b allows a clear comparison of the array before and after FEBIE. This was confirmed by AFM measurements (Figure 4) performed over the array containing lines spaced 150 nm apart. The red and blue plots represent the profile of the as-deposited array and the array post-cleaning, from which it is evident that the carbon has been successfully removed from the exposed areas. In one step, therefore, the lines have been separated and the halo around the deposit has been removed. 

**Figure 5:**
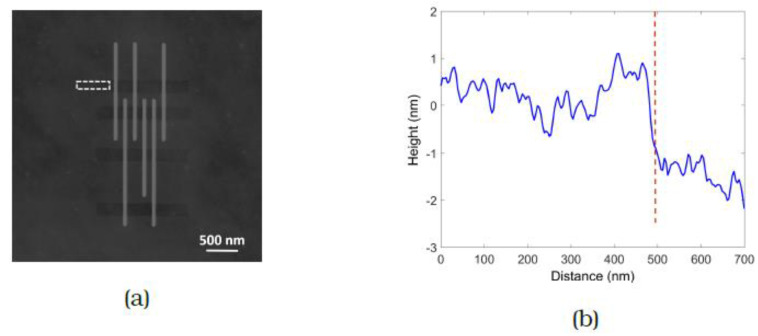
(**a**) AFM image with the dotted lines indicating an area at the edge of the etched region, where the thickness of the carbon film removed is measured from (**b**) to be about 1 nm.

**Figure 6:**
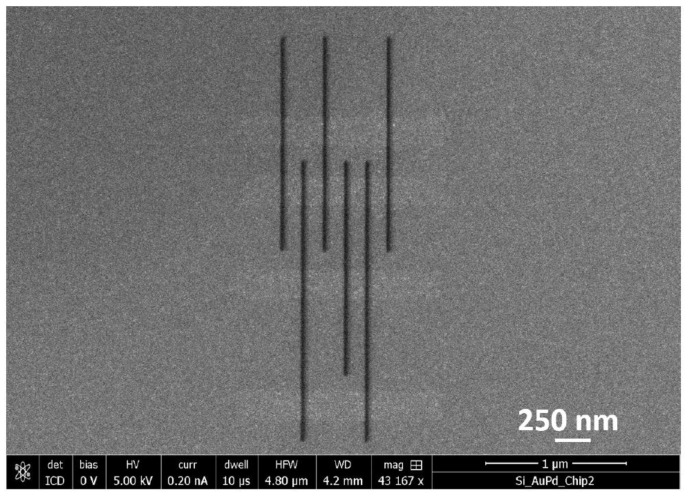
Backscattered electron (BSE) image of the array, also displaying a change in signal from the etched region. The thickness of the carbon film removed is reflected in the BSE contrast even for as little as 1 nm, meaning that BSE imaging would be a good method to monitor height changes. This is especially useful for lines at smaller spacing where height measurement of interconnecting material by AFM would not be accurate.

An AFM scan at the outer edge of the cleaned region (dotted lines in Figure 5) shows the thickness of the carbon film removed there to be as little as 1 nm. So, it is interesting to note that the BSE image of the array (Figure 6) also shows a change in contrast. Since no change in the crystal structure of the substrate is expected to occur here and other changes such as chemical modification of the surface would not affect the BSE contrast anyway, we conclude that BSE imaging using the ICD is highly sensitive to changes in height. This is useful when cleaning dense arrays where the spacing between lines is too small to allow reliable AFM measurements. The increase in BSE signal can be used as confirmation of successful carbon removal.

Recipe 2: High resolution FEBIE in between FEBID lines

**Figure 7:**
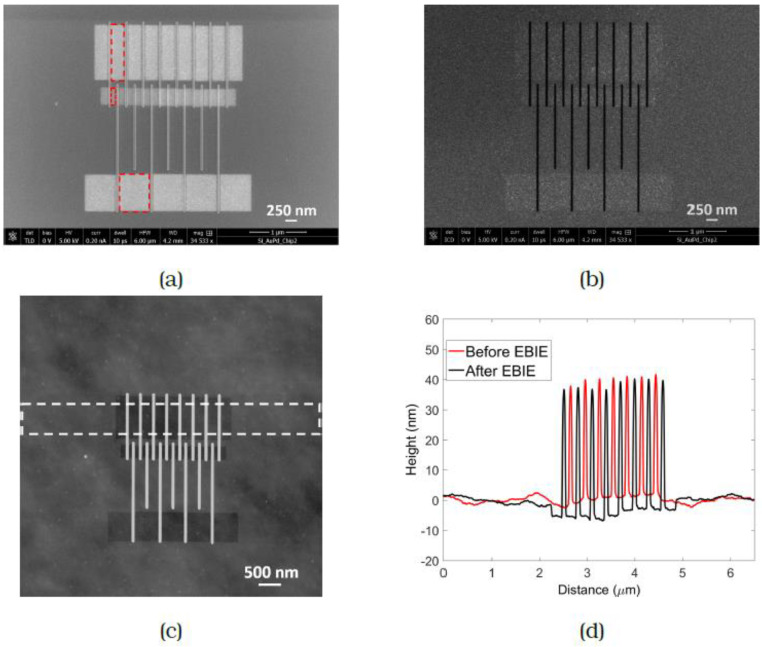
(**a**) SE (**b**) BSE and (**c**) AFM images of an array of carbon FEBID lines containing line spacings of 150 nm, 300 nm and 600 nm after high resolution FEBIE in between the lines. The dimensions of the area to be etched were determined by inspection of the through-lens SE detector (TLD) image and selected to just fill the gap between the lines as shown by the dashed black rectangles. (**d**) Integrated height plot of array of lines spaced 300 nm apart before and after cleaning, demonstrating successful removal of interconnects.

In an array of thicker FEBID lines, the connecting material might be thicker as well. Even for shallow lines, as the separation between the lines is reduced, the connecting material becomes relatively more significant. If such a high dose is required to clean the interconnects that the lines themselves would be damaged in the process, a large area etch is no longer an attractive technique. An experiment is described to remove material from the interconnects by positioning the patterns in between the lines. The dimensions of the etch pattern were determined by eye based on the TLD image, exposing the entire gap between the lines (as indicated by the dashed red rectangles in Figure 7a). The FEBIE parameters were the same as in the previous experiment and the dose was determined in the same manner. It was typically in the range of a few tens of C/cm^2^. Figure 7 shows the pattern that was cleaned, consisting of three sets of arrays with line spacing of 150 nm, 300 nm and 600 nm. FEBIE was carried out in serial mode from left to right. The SE (a) and BSE (b) images show brightening in the exposed regions and the integrated AFM line scan of one of the arrays in the pattern (spacing = 300 nm) shown in (c) provides a comparison of the line profile in the etched region with the as-deposited profile taken on the same array. The red and blue plots represent the as-deposited array and the array post-cleaning, as before, with a clear reduction in background deposit visible between the lines.

## 4. Discussion

**Figure 8:**
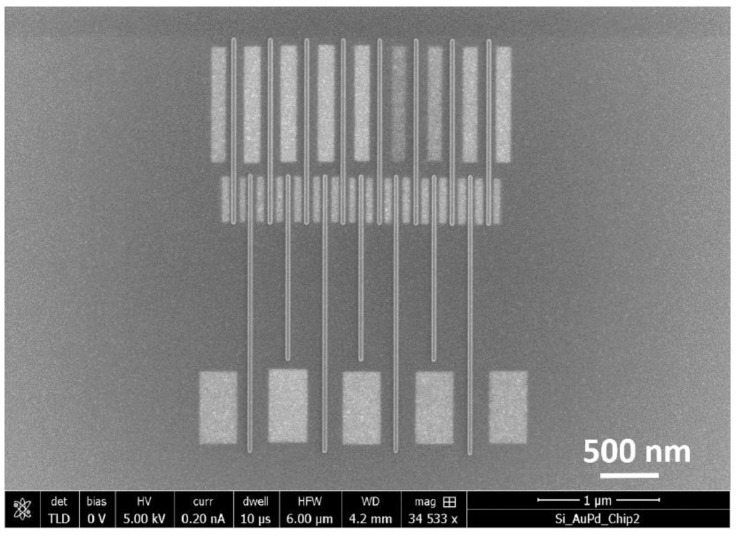
SE image of array identical to that in Figure 7 after etching in between the lines with higher resolution, making sure to leave the FEBID lines unaffected.

Focused electron beam-induced etching in a high vacuum SEM using water as a precursor has been applied to successfully remove interconnects in dense FEBID lines of carbon. The influence of etching dose and strategy have been analysed to enable the selection of the most suitable method. The effect of this exposure on the shape of dense FEBID lines has been studied using SE, BSE and AFM measurements and this can be used to tailor the cleaning technique. This result has broad applications in FEBID as several commonly used precursors contain carbon which remains in the deposit, giving rise to unwanted conducting channels between deposits. This technique ensures that although the metal particles in the halo would remain, the removal of conducting paths between them could be ensured by the removal of carbon. For lithography, it is of utmost importance to have well separated lines, and this has been shown to be possible using two recipes. Large area FEBIE of an entire field of view containing the lines is a fast and easy process that results in the successful removal of the interconnecting carbon film. The increase in SE signal upon removal of carbon, exposing the underlying gold-palladium substrate has been used to determine the electron dose needed for FEBIE. The height of the lines is somewhat affected by the exposure and for applications where this is undesirable, another technique has been proposed. By carefully positioning an etch pattern in between the lines using the SE image, it is possible to affect the same process, this time with no change to the height of the line. By further reducing the width of the etched area, it is possible to perform the same without affecting the bulk of the line. This allows the line width to remain intact—another advantage of this method. An identical pattern to the one described in Figure 7 was etched in this manner and Figure 8 shows that the technique is sufficiently well controlled to tune the extent of the etching. 

This would of course become increasingly difficult to perform at higher and higher resolution. This can be addressed by improving the resolution of the FEBIE process. As it is an electron beam-induced process, it has in principle similar resolution as FEBID. The problem arises because of the use of high current, which is necessary to suppress co-deposition of contamination. By working in a very clean system, ideally one where no precursor other than water is let in, and by following strict guidelines for samples that are allowed to be mounted in the SEM, it would be possible to perform FEBIE with a low current. A high-resolution beam could then be used to perform the etching in a very local manner with even more control. The time taken for the process would increase if the current were lowered, but since the doses used are quite low to begin with, this would remain an attractive cleaning technique. The use of the carbon precursor dodecane also presents some challenges since, upon continuous use over some hours, it appears to remain in the chamber for quite some time. While the sticking capability of dodecane is responsible for its high FEBID yield, this is not the limiting factor in high resolution experiments. Since the lines patterned would be narrower and contain less material, it would be beneficial to use a precursor that pumps out more quickly instead. Alternatively, another precursor such as oxygen could be used for FEBIE, avoiding this problem. Some modifications to the GIS would have to be implemented to ensure sufficient local flux of oxygen while maintaining high vacuum in the SEM chamber. Regular cleaning of the SEM using an oxygen plasma would also help in the maintenance of a clean environment for both FEBID and FEBIE. It should be pointed out here that the etching of other materials than carbon would require a precursor that volatilizes that particular deposit material. This work demonstrating the etching of carbon not only provides proof of concept of the technique, but it is also directly applicable to the removal of the carbonaceous halo surrounding FEBID deposits made with a variety of precursors.

## Data Availability

The data presented in this study are available on request from the corresponding author.
